# Idiopathic Myointimal Hyperplasia of Mesenteric Veins of the Ileum and Colon in a Patient with Crohn's Disease: A Case Report and Brief Review of the Literature

**DOI:** 10.1155/2017/6793031

**Published:** 2017-08-15

**Authors:** Sharon J. Song, Stuti G. Shroff

**Affiliations:** Department of Pathology and Laboratory Medicine, Hospital of the University of Pennsylvania, Philadelphia, PA 19104, USA

## Abstract

Idiopathic myointimal hyperplasia of the mesenteric veins (IMHMV) is a rare disease characterized by intimal smooth muscle proliferation, leading to the thickening of small to medium-sized mesenteric veins. This vascular disease mimics inflammatory bowel disease (IBD) clinically and endoscopically, while showing ischemic mucosal changes without the classic features of IBD on biopsy. Given the mixed picture, this entity is frequently misdiagnosed. Surgical resection of the diseased bowel segment reveals the true etiology of the pathology and is curative. We describe a case of a 59-year-old man with a long-standing history of Crohn's disease refractory to medical therapy and status after multiple small bowel resections. The patient underwent a subtotal abdominal colectomy with pathology showing dense, indurated mesenteric adipose tissue, significant muscularis propria hypertrophy, and myointimal hyperplasia of the mesenteric veins in a peri-ileal and pericolic distribution, as confirmed by elastin stain. No evidence of mucosal ischemic changes or findings of chronicity or acuity were seen. IMHMV, a rare disease with a mixed presentation, poses a significant diagnostic challenge to clinicians and pathologists.

## 1. Introduction

Idiopathic myointimal hyperplasia of the mesenteric veins (IMHMV) is a poorly understood disease that poses a diagnostic challenge to clinicians and pathologists. Clinically and endoscopically, patients appear to have inflammatory bowel disease (IBD); however, biopsies show ischemic abnormalities without the classic features of IBD. Patients have a relatively protracted clinical course refractory to medical treatment, and it is not until the diseased bowel segment is resected that histology reveals the underlying etiology. IMHMV is characterized by thickened small and medium-sized mesenteric veins with the hallmark feature of intimal smooth muscle proliferation, leading to luminal occlusion and mucosal ischemic changes. Herein, we present a case of IMHMV in a 59-year-old man with a long-standing history of Crohn's disease.

## 2. Case Presentation

A 59-year-old man with a 30-year history of Crohn's disease was referred to our institution for ongoing symptoms of bloating, abdominal pain, and alternating bouts of constipation and diarrhea with occasional incontinence despite treatment with infliximab and azathioprine. He had three prior small bowel resections at the ileocecal junction for intestinal obstruction with the last surgery performed 15 years ago. The two prior resections, according to pathology reports available for review, demonstrated changes consistent with Crohn's disease, including transmural inflammation and multiple inflammatory pseudopolyps.

His most recent colonoscopy at the outside institution showed patchy mild inflammation from the rectum to descending colon; however, surveillance was limited by adhesions and strictures preventing passage of the colonoscope proximal to 50 cm. Endoscopic biopsies obtained every 10 cm did not reveal any active disease or dysplasia. A CT scan showed diffuse mucosal thickening and edema from the rectum to descending colon with edema of the pericolic fat. MR enterography subsequently performed at our institution revealed minimally active inflammatory disease from the rectosigmoid to descending colon without any evidence of complications or active disease involving the neoterminal ileum.

**Figure 1:**
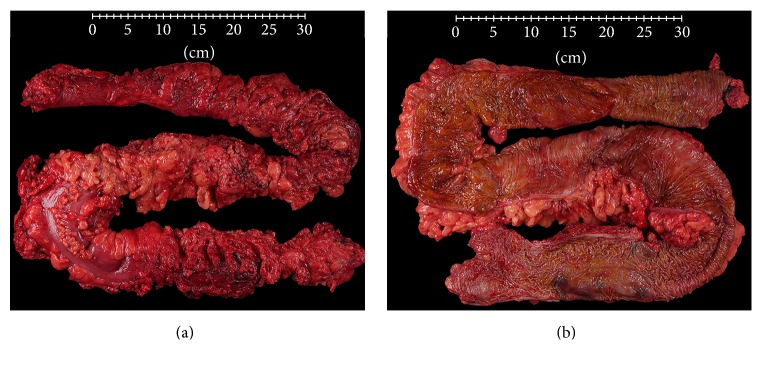
Gross examination of the ileocolonic surgical resection specimen revealed extensive fat wrapping and induration of the peri-ileal and pericolonic adipose tissue (a). Examination of the mucosal surface was relatively unremarkable without evidence of strictures, fistulae, or mucosal ulcers (b).

**Figure 2:**
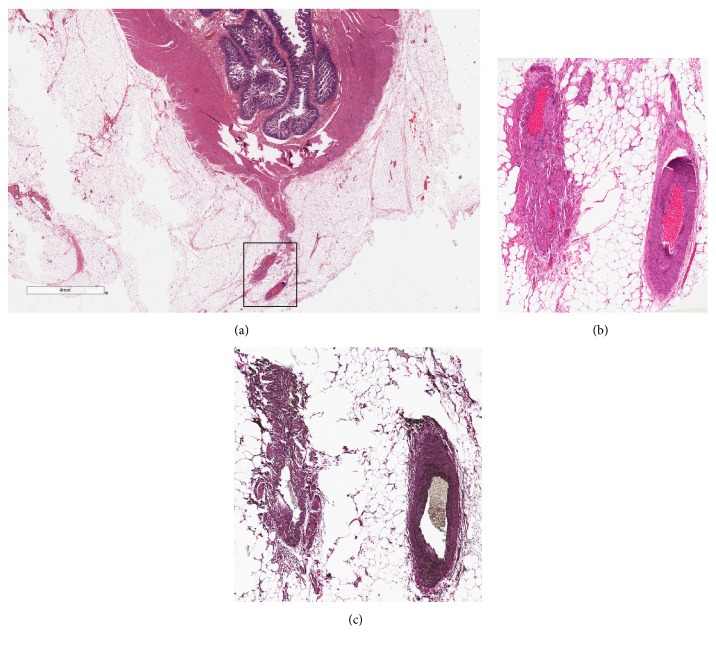
Involvement of the neoterminal ileum by idiopathic myointimal hyperplasia of the mesenteric veins. A representative low-magnification view of the ileum revealed an unremarkable mucosa and ileal wall (a). On high magnification, the peri-ileal veins demonstrated myointimal hyperplasia (b) as evidenced by elastin staining (c).

Due to nonresolution of his symptoms a decision was then made to proceed with an open subtotal abdominal colectomy with end ileostomy and Hartmann's pouch formation. Grossly, the specimen received was composed of a portion of ileum anastomosed to a markedly dilated colon, including the sigmoid colon, and notable for extensive fat wrapping associated with dense, indurated mesenteric adipose tissue, particularly in the mid and distal colon (Figure 1(a)). The wall of the ileum and colon were thickened and edematous but revealed an otherwise unremarkable mucosal surface with no strictures, ulcerations, fissures, fistulas, pseudopolyps, or perforations (Figures 1(b) and 2(a)). Gross examination of the resection specimen was also significant for soft submucosal nodules scattered throughout the colon.

**Figure 3:**
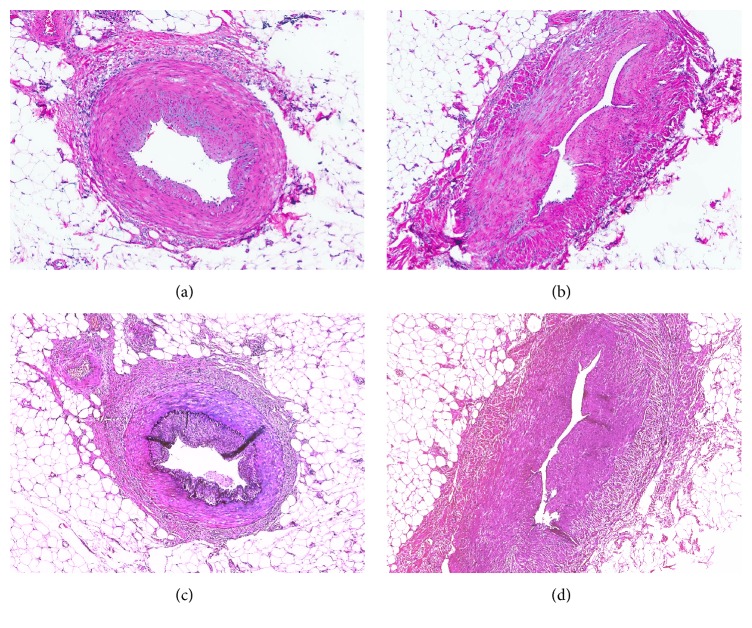
Paired H&E (a, b) and elastin (c, d) stained high-magnification images of mesenteric vessels in the pericolonic soft tissue. Elastic laminae, visible in the artery (a, c), are absent from the vein (b, d), which shows myointimal hyperplasia.

Histologic evaluation revealed myointimal hyperplasia of the mesenteric veins in the peri-ileal (Figure 2) and pericolic (Figure 3) soft tissue as confirmed by elastin stain as well as significant muscularis propria hypertrophy. Absent were the classic findings of chronicity (mucosal crypt architectural distortion, left-sided Paneth cell metaplasia, transmural chronic inflammation, fistulas, and fissures), and acuity (cryptitis, crypt abscesses, and ulcerations) associated with Crohn's disease and mucosal ischemic changes typically seen with IMHMV. Additionally, there was no evidence of small or large vessel vasculitis. The scattered submucosal nodules were revealed to be lipomas.

Postoperatively, the patient made a gradual recovery and was discharged 13 days after surgery. No additional medical management was needed.

## 3. Discussion

Mesenteric ischemia is most commonly caused by arterial thromboembolic disease and much less commonly by venous occlusion, which, when present, is usually due to venous thrombosis [1]. Idiopathic myointimal hyperplasia of the mesenteric veins is a rare, poorly understood nonthrombotic and noninflammatory vascular disease that mimics IBD. First described in 1991 by Genta and Haggitt, over 22 cases of IMHMV have been reported since that time (Table 1) [2]. This number likely underestimates the true incidence of IMHMV given that biopsies often show nonspecific findings and the condition is routinely diagnosed after histologic review of the resected specimen (only one case to date in the literature was diagnosed preoperatively) [3]. Hence, milder cases that do not require surgical resection may be misdiagnosed.

Classically, IMHMV was thought to be a disease of the rectosigmoid colon affecting young healthy males [4]. Review of the current literature, however, shows the median age at time of diagnosis to be 59 years. Men are more likely to be affected than women by 2.7 : 1 with six of the reported cases involving women [3, 5–9]. Additionally, although the majority of cases involve the rectosigmoid colon, numerous cases now have noted extension of the disease proximally along the descending colon as well as involvement of the jejunum [5], ileum [8–10], and entire colon sparing the rectum [11].

The clinical and endoscopic impression is that of IBD in contrast to mucosal biopsy findings, which are more suggestive of an ischemic etiology [4]. Patients typically present with weight loss, abdominal pain, and bloody diarrhea [12, 13]. Colonoscopy may show a friable mucosa with ulcerations and erythematous and edematous changes [14–16]. Full thickness sections show the pathognomonic histologic feature of arterial-sparing intimal smooth muscle hyperplasia of small- and medium-sized mesenteric veins, which can be confirmed by an SMA stain. Additionally, an elastin stain can be used to highlight the elastic laminae present in arteries and absent in the affected veins, an important diagnostic clue [12]. The degree of luminal occlusion is variable but often results in an ischemic pattern of injury such as ulceration, superficial withering of crypts, edema, congestion, and, later on in the disease process, hyalinization and fibrosis of the lamina propria and/or submucosa [3, 6, 11, 17]. Notably absent on histology are chronic inflammatory changes.

In terms of treatment, patients diagnosed with IMHMV have often endured multiple rounds of failed medical therapy for IBD, leading to surgical resection of the diseased bowel. Segmental resection is curative and follow-up for up to 7 years has been uneventful with no recurrence of disease [12].

The case we report here is unique in several ways. To our knowledge, this is the first case of IMHMV with involvement of the neoterminal ileum to sigmoid colon in a patient with a long-standing clinicopathologic history of Crohn's disease. Unlike other cases where time to surgery has ranged from 1 month to 2 years and clinical impression often is that of IBD or ulcerative colitis, this patient presented to our institution with a 30-year history of pathologically demonstrated Crohn's disease. Interestingly, despite the patient's experience of progressively worsening symptoms and the degree of venous occlusion present in his bowel, the resected specimen revealed a normal-appearing mucosa without any ischemic changes. Additionally absent were the classic findings of chronicity and acuity typically associated with Crohn's disease.

The etiology of IMHMV is unknown and pathophysiology remains speculative. The vessel abnormalities in IMHMV have been reported to resemble histologically failed saphenous vein grafts from patients that have undergone aortocoronary bypass surgery [18] as well as stenotic arteriovenous fistulas in dialysis patients [19], suggesting an “arterialization” of veins due to increased intraluminal pressure. Some have proposed that the mobility of the sigmoid colon renders it susceptible to traumatic injury secondary to torsion, volvulus, or stretch, leading to arteriovenous fistulization and ultimately myointimal hyperplasia of the mesenteric veins [18, 20]. However, this mechanism fails to account for the occurrence of IMHMV in other parts of the bowel that lack such mobility and further investigation into the pathogenesis is needed.

## 4. Conclusion

IMHMV is a challenging diagnosis to make, given the discordant clinical, endoscopic, and biopsy findings. As more cases are reported, the demographics for this entity are also broadening and it is important to keep this condition in the differential for patients with inconsistencies in their presentation. In cases of biopsies showing ischemic changes and blood vessel abnormalities in an anatomic distribution incongruent with ischemic colitis, negative angiography may help point towards a diagnosis of IMHMV [3]. Increased awareness of this disease among clinicians and pathologists may help with earlier recognition of it and ultimately better management of affected patients.

## Figures and Tables

**Table 1:** Reported features of Idiopathic myointimal hyperplasia of mesenteric veins.

Year	Authors	Age/sex	Affected site	Clinical impression	Time to surgery	Follow-up
2017	Song et al.	59 M	Sigmoid to ileum	Crohn's	30 yr	NA
2016	Guadagno et al.	59 F	Ileum	IBD, NET	6 mo	3 yr
2016	Yun et al.	64 M	Rectum to distal transverse	UC	2 yr	6 mo
2015	Wangensteen et al.	62 F	Rectosigmoid	IMHMV	2 mo	>1.5 yr
2015	Sahara et al.	76 M	Rectosigmoid	IBD, ischemia	1 yr	NA
2015	Abbott et al.	58 M	Rectum to descending colon	Colitis, ischemia	NA	NA
2015	Laskaratos	62 F	Ileum	Inflammation, ulceration	NA	NA
2014	Korenblit et al.	59 M	Rectosigmoid	Colitis	1 mo	3 mo
2013	Thomas [21]	62 M	Rectosigmoid	Colitis	1 mo	NA
2013	Feo et al.	75 F	Rectosigmoid	Ischemic colitis	>6 mo	No recurrence
2012	Chiang et al.	60 M	Rectosigmoid	UC	2 mo	4 mo
2012	Lanitis et al.	81 M	Ileum	Not reported	6 mo	NA
2012	Korenblit et al.	62 M	Entire colon (rectal sparing)	UC	18 mo	4 d
2011	García-Castellanos et al.	32 F	Rectum to splenic flexure	Pneumatosis intestinalis, colon ulceration	3 mo	24 mo
2006	Sherman et al.	38 M	Rectosigmoid	IBD	5 mo	18 mo
1999	Savoie and Abrams	22 M	Rectosigmoid	UC	NA	10 mo
1998	Bryant	42 F	Jejunum	Unknown	NA	NA
1996	Abu-Alfa et al.	58 M	Sigmoid	IBD, ischemic colitis	>1 yr	NA
1991	Genta and Haggitt	30 M	Sigmoid	Stricture	1 mo	7 yr
1991	Genta and Haggitt	38 M	Sigmoid to descending colon	UC	2 mo	1 yr
1991	Genta and Haggitt	25 M	Rectosigmoid	UC	>6 mo	4 yr
1991	Genta and Haggitt	67 M	Sigmoid	Crohn's	3 mo	2 yr
